# Freezing of Gait in Parkinson’s Disease Patients Treated with Bilateral Subthalamic Nucleus Deep Brain Stimulation: A Long-Term Overview

**DOI:** 10.3390/biomedicines10092214

**Published:** 2022-09-07

**Authors:** Giulia Di Rauso, Francesco Cavallieri, Isabella Campanini, Annalisa Gessani, Valentina Fioravanti, Alberto Feletti, Benedetta Damiano, Sara Scaltriti, Elisa Bardi, Maria Giulia Corni, Francesca Antonelli, Vittorio Rispoli, Francesca Cavalleri, Maria Angela Molinari, Sara Contardi, Elisa Menozzi, Annette Puzzolante, Jessica Rossi, Stefano Meletti, Giuseppe Biagini, Giacomo Pavesi, Valerie Fraix, Mirco Lusuardi, Alessandro Fraternali, Annibale Versari, Carla Budriesi, Elena Moro, Andrea Merlo, Franco Valzania

**Affiliations:** 1 Neurology Unit, Neuromotor & Rehabilitation Department, Azienda USL-IRCCS di Reggio Emilia, 42123 Reggio Emilia, Italy; 2 Neurology Unit, Neuroscience Head Neck Department, Ospedale Civile Baggiovara (OCB) Hospital, Azienda Ospedaliero-Universitaria di Modena, 41126 Modena, Italy; 3 Department of Biomedical, Metabolic and Neural Sciences, University of Modena and Reggio Emilia, 41125 Modena, Italy; 4 Clinical and Experimental Medicine PhD Program, University of Modena and Reggio Emilia, 41125 Modena, Italy; 5 LAM-Motion Analysis Laboratory, Neuromotor and Rehabilitation Department, Azienda USL-IRCCS di Reggio Emilia, S. Sebastiano Hospital, 42015 Correggio, Italy; 6 Department of Neurosciences, Biomedicine, and Movement Sciences, Institute of Neurosurgery, University of Verona, 37124 Verona, Italy; 7 Neurosurgery Unit, Azienda Ospedaliero-Universitaria of Modena, Ospedale Civile Baggiovara (OCB) Hospital, 41126 Modena, Italy; 8 Division of Neuroradiology, Department of Neuroscience, Nuovo Ospedale Civile S. Agostino Estense, 41126 Modena, Italy; 9 IRCCS Istituto delle Scienze Neurologiche di Bologna, Department of Neurology and Stroke Center, Maggiore Hospital, 40133 Bologna, Italy; 10 Department of Clinical and Movement Neurosciences, UCL Queen Square Institute of Neurology, London WC1N 3BG, UK; 11 Univ. Grenoble Alpes, Inserm, U1216, CHU Grenoble Alpes, Grenoble Institut Neurosciences, 38000 Grenoble, France; 12 Neuromotor and Rehabilitation Department, Azienda USL-IRCCS di Reggio Emilia, 42015 Reggio Emilia, Italy; 13 Nuclear Medicine Unit, Azienda Unità Sanitaria Locale-IRCCS of Reggio Emilia, 42100 Reggio Emilia, Italy

**Keywords:** axial symptoms, deep brain stimulation, freezing, gait, long-term, Parkinson’s disease, STN-DBS, subthalamic nucleus

## Abstract

Bilateral subthalamic nucleus deep brain stimulation (STN-DBS) is an effective treatment in advanced Parkinson’s Disease (PD). However, the effects of STN-DBS on freezing of gait (FOG) are still debated, particularly in the long-term follow-up (≥5-years). The main aim of the current study is to evaluate the long-term effects of STN-DBS on FOG. Twenty STN-DBS treated PD patients were included. Each patient was assessed before surgery through a detailed neurological evaluation, including FOG score, and revaluated in the long-term (median follow-up: 5-years) in different stimulation and drug conditions. In the long term follow-up, FOG score significantly worsened in the off-stimulation/off-medication condition compared with the pre-operative off-medication assessment (z = −1.930; *p* = 0.05) but not in the on-stimulation/off-medication (z = −0.357; *p* = 0.721). There was also a significant improvement of FOG at long-term assessment by comparing on-stimulation/off-medication and off-stimulation/off-medication conditions (z = −2.944; *p* = 0.003). These results highlight the possible beneficial long-term effects of STN-DBS on FOG.

## 1. Introduction

Parkinson’s Disease (PD) is the second most common neurodegenerative disorder after Alzheimer’s disease and is the most common type of parkinsonism [1]. Cardinal manifestations of PD include bradykinesia, rest tremor and rigidity [1]. PD axial symptoms include dysarthria, dysphagia, gait and postural disorders, and are often one of the main causes of long-term impairment and disability in PD patients [2]. Particularly, gait disturbances in PD are characterized by slowness and difficulty in starting walking, reduction or asymmetry of arm swing, gait festination and FOG [3,4]. FOG is characterized by difficulty in gait initiation or sudden inability to keep moving forward [5]. Three different clinical phenotypes of FOG have been distinguished: 1. tremor in place with rapid and alternating movements of the knees (knee trembling); 2. shuffling forward with very short, grounded steps; 3. complete akinesia with no movement [6]. Moreover, some types of FOG improve with levodopa or subthalamic nucleus (STN) stimulation (levodopa-responsive FOG), whereas others do not (levodopa-resistant FOG) or even get worse with long-lasting levodopa therapy [5,7]. Different conditions may favor the appearance of FOG: the turning phase during walking, the beginning of walking, the overcoming of obstacles or the crossing of narrow paths [6]. Finally, the execution of other activities during walking (dual task condition) carries a greater risk of developing FOG or falling [5]. The intensity of the different cardinal and axial symptoms may vary in the three main motor phenotypes: Postural Instability Gait Disorder phenotype (PIGD), tremor-dominant phenotype and indeterminate phenotype [8]. 

STN-DBS represents a short and long-term effective treatment in advanced PD patients [9,10,11,12,13]. In the long-term, STN-DBS allows a stable improvement of motor complications, tremor and rigidity but with a less relevant effect on axial symptoms (e.g., gait and balance symptoms, speech and swallowing troubles) and cognitive decline [10]. However, few data about the evolution of FOG in long term follow up after bilateral STN-DBS are available mostly based on the gait and FOG items of the Unified Parkinson’s Disease Rating Scale (UPDRS) and the Movement Disorder Society-Sponsored Revision of the UPDRS (MDS-UPDRS) [14]. This had limited the quantification of the severity and the phenotyping of FOG in the long-term after surgery. The aim of this study is to assess the long-term effects of bilateral STN-DBS on FOG in a cohort of advanced PD patients with a standardized clinical approach. 

## 2. Materials and Methods

### 2.1. Participants

This observational study included all consecutive patients who underwent bilateral STN-DBS from 2012 to 2018 at the Neurological Unit of the OCB University Hospital, Modena, Italy. At time of surgery all patients fulfilled the diagnosis of PD according to the UK Brain Bank criteria [15], complained of disabling motor complications not well controlled with dopaminergic medications and presented an age at surgery younger than 70 years. The presence of severe cognitive or psychiatric disorders, severe atrophy, or diffuse cerebral ischemic lesions on brain magnetic resonance imaging (MRI) and systemic comorbidities interfering with surgery represented the exclusion criteria. The study was approved by the Ethics Committee of the Area Vasta Emilia Nord N° 2019/0056629, 14 May 2019.

### 2.2. Clinical Assessment

Patients were evaluated in accordance with the Core Assessment Program for Surgical Interventional Therapies in Parkinson’s Disease (CAPSIT-PD) [16]. Pre-operative demographic, clinical and neuroimaging variables were collected by reviewing medical records. At pre-operative evaluation, disease stage was quantified with the Hoehn and Yahr scale (H&Y), and the motor severity of the disease through the Unified Parkinson’s Disease Rating Scale (UPDRS) part III total score in the “off-medication condition” (obtained after a 12-h antiparkinsonian medication withdrawal) and in the “on-medication” condition (obtained 60 min after the administration of a 30% higher dose of the usual levodopa morning intake) [17]. Several sub-scores from the UPDRS were calculated including for akinesia, tremor and postural instability/gait disorders (PIGD). PD motor phenotype (tremor dominant [TD], indeterminate and PIGD) was obtained using a previously published algorithm [18]. Particularly, PIGD sub-score was obtained by the UPDRS-II items 13–15 and the UPDRS-III items 29 and 30 (score 0–20), while tremor sub-score was calculated from the UPDRS-II items 16 and the UPDRS-III items 20 and 21 (score 0–32). Moreover, patients were divided into three motor phenotypes by calculating the ratio between tremor related sub-scores compared to postural instability/gait disorders sub-scores. Based on the ratio, the patients were classified into tremor dominant phenotype (ratio > 1.5), PIGD phenotype (ratio < 1) and indeterminate phenotype (ratio between 1.0 and 1.5) [18]. FOG was evaluated and quantified through the FOG score [19]. This consists of a four-level interval scale applied in four different FOG provocative conditions: start to walk, clockwise and counter-clockwise turning and passage through doorway. In each situation, zero points were given in the absence of FOG or festination, one point in the presence of festination or FOG with shuffling steps and two points for the observation of akinetic FOG or FOG with trembling-in-place legs. Finally, three points were given for any abortion of the task or when patients needed the examiner interference to overcome the FOG episode (e.g., acoustic clue). Usually, the patients were asked to perform three consecutive levels of multiple tasking: the first time with no additional task (only walking), the second time performing a second motor task and the third time with both a motor and a mental task. The FOG score resulted from the addition of the 12 items rated during the performance of the four situative maneuvers at three levels of dual-tasking ranging from 0 to 36 points [19]. In our study, we tested only the first condition without any additional task, so the FOG score ranges from 0 (absence of FOG or festination) to 12 (impossibility to perform passage without help). All patients underwent a 3 Tesla brain MRI in order to evaluate the presence of white matter hyperintensities of vascular origin. Patients were also screened for the presence of mutations in the Leucine-rich repeat kinase 2 (LRRK2), Glucocerebrosidase (GBA), Alpha synuclein (SNCA) and PARKIN (PRKN) genes. The total amount of dopaminergic medications was calculated as levodopa equivalent daily dose (LEDD) in milligrams (mg) as previously reported [20]. All subjects were re-evaluated in the long-term after surgery. Neurological evaluations were superimposable with pre-operative ones and were performed in the same day in different pharmacological and stimulation conditions including, firstly, on-stimulation/off-medication (washout of at least 12 h of dopaminergic medications); secondly, off-stimulation/off-medication (stimulation was temporarily turned off for at least one hour); and finally, on-stimulation/on-medication (stimulation was turned on and dopaminergic therapy was administered (early morning LEDD plus 30%)). All patients underwent a detailed neuropsychological assessment both before surgery and at long-term evaluation including phonemic fluency, spatial perception (localization of numbers), Raven’s progressive matrices, Stroop test and Trail making test part B. Furthermore, a blinded perceptual and acoustic speech evaluation was performed both pre-operatively and in the post-operative assessment by two speech therapists with certified experience in movement disorder-related speech disturbances. In particular, word intelligibility was obtained by calculating the percentage of 25 recorded words correctly transcribed by the examiner.

### 2.3. Statistical Analysis

Descriptive statistics were used to describe demographic characteristics, baseline and short- and long-term clinical data. Continuous variables were expressed as mean (±standard deviation [SD]) and median (range), whereas frequency and percentage were calculated for categorical variables. The primary objectives of the study were: to assess the evolution of FOG due to disease progression without any kind of treatment, obtained by comparing the total FOG score in the off-stimulation/off-medication condition at long-term evaluation with the pre-operative off-medication condition; to assess the long-term effects of bilateral STN-DBS on FOG evaluated by comparing the total FOG score in the on-stimulation/off-medication condition at long-term evaluation with the off-stimulation/off-medication condition. Furthermore, the total FOG score in the on-stimulation/off-medication condition was compared to the baseline score in the off-medication assessment. Based on the presence of long-term post-operative worsening of FOG total score, patients were divided into two groups defined as “stable” and “worsened”. Due to the limited samples, statistical analysis between the two subgroups was not performed. Secondary objectives included the variations in the total FOG score in the other different pre-operative and post-operative conditions tested. The presence of significant differences in FOG total score in the different conditions tested was calculated using the Friedman test with subsequent post-hoc Wilcoxon signed rank test. Finally, to find if other symptoms ameliorated in the “stable” subgroup, we performed the Friedman test with subsequent post-hoc Wilcoxon signed rank test. A *p*-value < 0.05 was considered significant. Statistical analysis was performed using the IBM SPSS Statistics for Windows version 20.0 (IBM, Armonk, NY, USA).

## 3. Results

### 3.1. Description of the Cohort

Twenty-five PD patients treated with bilateral STN-DBS at the Neurology Unit of the OCB Hospital, Italy, from 1 January 2012 to 31 December 2018, were included and recruited from September 2019 to October 2021. Data from five patients were not analyzed because of pre-operative missing information. Table A1 lists the main pre-operative demographic and clinical characteristics of the cohort (males: 16; disease duration at surgery: 9.70 (±4.88) years; age at surgery: 58.85 (±6.12) years; age at PD onset 48.85 (±5.53) years). Pre-operative brain MRI showed the presence of mild white matter hyperintensities of vascular origin in three patients (15%). Fifteen patients (75%) were included in the PIGD subtype, four in the indeterminate subtype (20%) and one in the TD subtype (5%). Genetic assessment revealed heterozygous mutation in the GBA gene in two patients (10%). The mean pre-operative levodopa responsiveness was 62.25% (±17.06%). Before surgery, 9/20 patients (45%) presented FOG in the off-medication state with 3.55 (±4.90) mean FOG total score. In the on-medication state, only 2/20 patients (10%) showed FOG with a mean total FOG score of 0.40 (±1.23). All patients were reevaluated with a median 5-years follow-up after surgery. Table A2 lists the main demographic and clinical characteristics of the cohort at post-operative assessment. Particularly, after surgery 13/20 patients (65%) presented FOG episodes in the off-stimulation/off-medication condition with 5.70 (±4.94) mean FOG total score; 9/20 patients (45%) presented FOG in the on stimulation/off medication condition and only 5/20 (25%) in the on-stimulation/on-medication condition. Moreover, in the long-term follow-up, the distribution of motor phenotypes consisted of 17 patients included in the PIGD subtype (85%), two in the indeterminate subtype (10%) and one in the TD (5%). Finally, the mean levodopa therapy duration at the evaluation was 11.80 (±2.88) years. 

Table A3 shows the main neuropsychological features. 

### 3.2. Primary Objectives

In the long term follow up, there was a significant worsening of total FOG score in the off-stimulation/off-medication condition compared to the pre-operative off-medication assessment (z = −1.930; *p* = 0.05). Furthermore, total FOG score significantly worsened in the off stimulation/off medication condition if compared with the on-stimulation/off-medication condition (z = −2.944; *p* = 0.003). On the contrary, FOG score did not significantly worsen in the post-operative on-stimulation/off-medication condition if compared with the pre-operative off-medication condition (z = −0.357; *p* = 0.721). Table A4 summaries changes of FOG total score and sub-scores in the different conditions.

### 3.3. Secondary Objectives

Fifteen patients were included in the “stable” subgroup and the remaining five patients in the “worsened”. Patients in the latter subgroup tended to show a greater pre-operative motor disease severity in on-state quantified by higher scores in the UPDRS part III total score and akinesia sub-score. Furthermore, they appeared to show a higher axial burden in all the post-operative conditions quantified through the PIGD sub-score (Table A5). In addition, both the akinesia sub-score in the on-stimulation/on-medication condition and the total FOG score in the on-stimulation/off-medication condition were generally higher in the “worsened” subgroup. On the contrary, disease duration, age at surgery, follow-up duration and stimulation parameters (see Table S1 in supplementary material) seemed not to be different between the two subgroups. Furthermore, patients included in the “stable” subgroup showed a significant worsening of total UPDRS-III total score (z = −3.411; *p* = 0.001), H&Y stage (z = −2.831; *p* = 0.005), akinesia (z = −3.413; *p* = 0.001), tremor (z = −2.965; *p* = 0.003) and PIGD (z = −3.126; *p* = 0.002) sub-scores in the off-stimulation/off-medication condition if compared with the on-stimulation/off-medication condition, meaning that the ”stable” subgroup showed a globally significant motor response to bilateral STN-DBS even in the long-term after surgery. 

By comparing the different conditions tested, several significant changes were found as reported in Table A4. Particularly, levodopa intake had a significant beneficial effect on FOG both at baseline (pre-operative off-state vs pre-operative on-state (*p* = 0.007)) and in the long-term after surgery (on-stimulation/off-medication vs on-stimulation/on-medication condition (*p* = 0.001)). On the contrary STN-DBS was not effective in the ON-FOG in the long-term. Finally concerning the different clinical phenotypes of FOG, we did not observe any particular trend in the effects of STN-DBS in knee-trembling, shuffling grounded steps and complete akinesia patients.

## 4. Discussion

The present study evaluated the long-term effect of bilateral STN-DBS on FOG in 20 patients with advanced PD using clinical measures. Overall, our results highlight that: (1) in the natural course of PD, as expected, FOG worsened in the absence of stimulation or dopaminergic treatment; (2) in the long-term follow-up, STN-DBS improved FOG. Furthermore, the worsening of FOG was not significant in the off-medication/on-stimulation condition compared to pre-operative off-state assessment thanks to the compensatory role of STN-DBS; (3) in the long-term follow up levodopa treatment continued to have some positive effect on FOG.

FOG occurs commonly in advanced Parkinson’s disease [6,21] and can even be documented in the early stage of PD. However, in this condition, FOG is typically mild and of short duration [6]. Several previous studies have documented that bilateral STN-DBS may improve FOG up to one year after surgery, in the same way as pre-operative drug therapy [5,22,23,24,25]. However, several studies have also shown that FOG can improve after surgery only if present in the off-medication condition before surgery [26]. Unfortunately, there is already a deterioration in axial symptoms five years after the intervention [27]. Indeed, data from long-term studies have confirmed that STN-DBS initially improves the UPDRS PIGD sub-score in the on-stimulation/off-medication condition, even if the score worsens over time until it reaches or exceeds pre-operative values eight years after surgery [26]. However, one of the main limitations of these studies was that the quantification of FOG was solely based on the UPDRS and not on a specific FOG scale. 

In order to find specific clinical characteristics associated with long-term FOG worsening, patients were divided into two groups, “stable” and “worsened”. Patients were significantly phenotyped, including clinical, genetic, neuropsychological and speech variables. Particularly, patients in the worsened subgroup tended to show a greater motor disease severity in the pre-operative on-state and in the post-operative on-stimulation/on-medication condition and seemed to have a major axial impairment. These results were in line with previous studies that have showed that, after STN-DBS, FOG severity is related to pre-operative FOG severity and global axial burden [28,29,30]. This data suggests that the beneficial effect of STN-DBS on FOG is not proportional to the pre-operative FOG severity. As a consequence, in patients with a pre-operative severe axial impairment, STN-DBS does not provide a significant clinical improvement in FOG. On the contrary, STN-DBS effect is remarkable in patients without a pre-operative harsh axial deterioration. 

Moreover, bearing in mind the important limitation of the small sample size, it is interesting to note that we did not find different incidence of GBA mutations in our cohort, even if it is well known that GBA-PD patients present a higher axial and cognitive burden if comparted with idiopathic PD [31]. Finally, patients in the worsened group seemed not to show worse cognitive or speech performances if compared with the “stable” group. Obviously future studies with a greater sample size will be needed to investigate this finding. Indeed, a direct correlation between FOG, speech disturbance, postural instability and cognitive decline, particularly executive dysfunction, has been previously reported [29,30,32]. Levodopa had a positive effect on FOG both at baseline and in the long-term after surgery. This data agrees with a previous study [33] conducted on 19 PD patients who were on chronic levodopa treatment and who experienced marked off-state FOG. They were tested by UPDRS part III and Hoehn and Yahr scale during off-state and approximately 1 h after their regular morning dose of levodopa. According to this study, all types of FOG improved significantly with levodopa treatment; particularly, levodopa significantly decreased the frequency and the akinetic type of FOG, with a tendency for shorter FOG episodes. It has been suggested that the specific dopamine receptor subtype (D1) had a crucial role in the effectiveness of levodopa on FOG [33]. In contrast, other studies highlighted an increase in FOG frequency in association with levodopa treatment [34,35]. It is well-known that some patients presenting off-state FOG (levodopa-responsive FOG) could develop on-state FOG over the years that becomes worse with levodopa [36]. However, in our opinion, we can assume that levodopa could not be responsible for the deterioration of FOG seen in our patients, which could be mainly due to disease progression. Finally, it is well known that FOG can occur during both off- and on-medication states [32] and it appears more frequently in the “off” pharmacological state and less frequently during the “on” phase [30,33]. Our study confirmed this data. 

The present study has several limitations including the lack of definition of the electrodes position and the small sample size. However, the latter limit was commonly shared by previous studies which tested patients in a long-term follow-up [37]. Another limit is that there has been no gold standard for FOG evaluation or quantification [37]. In fact, the FOG questionnaire, the first validated tool to assess FOG, is a subjective assessment. Despite this limit, we tried to analyze and quantify FOG systematically, applying an objective score to a videorecording. However, since we did not ask the patients whether they had subjectively experienced FOG, we could have missed some brief and subclinical episodes. 

## 5. Conclusions

In the present study, we analyzed the long-term effects of bilateral STN-DBS on FOG in a cohort of advanced PD patients. Our data confirm that PD progression led to a significant worsening of FOG, tested without any treatments. Against this background, bilateral STN-DBS was effective in controlling the worsening of FOG over time, as demonstrated either by the stability of the FOG score compared to the pre-operative baseline condition or by the worsening of the FOG score during the off-stimulation condition. Patients who experienced a long-term worsening of FOG showed a greater pre-operative motor disease severity and had a more severe axial impairment in all the post-operative conditions, suggesting the importance of prompt surgery. Finally, levodopa treatment also had a long-term positive effect on FOG. 

FOG leads to a marked increase in the risk of falling in PD patients and represents an independent factor of a worsening of their quality of life [38]. From this point of view, further studies on FOG are needed in order to confirm our results and to better elucidate the role of STN-DBS on FOG.

## Data Availability

The data that support the findings of this study are available from the corresponding author, I.C., upon reasonable request.

## Supplementary Materials

The following supporting information can be downloaded at: https://www.mdpi.com/article/10.3390/biomedicines10092214/s1, Table S1: Stimulation parameters of the cohort.

## Appendix A

**Table A1:** Pre-operative demographic and clinical characteristics.

Variable	No. (%); Mean [±SD]; Median {Range}
**Sex** Male Female	16 (80.00%) 4 (20.00%)
**Brain MRI: presence of WMH** No Yes	17 (85.00%) 3 (15.00%)
**PD motor subtype** PIGD Indeterminate TD	15 (75.00%) 4 (20.00%) 1 (5.00%)
**Genetic analysis** Negative Positive	18 (90.00%) 2 (10.00%)
**PD duration, yr**	9.70 [±4.88]; 8.00 {5.00–25.00}
**Age at surgery, yr**	58.85 [±6.12]; 60.00 {46.00–71.00}
**Age at PD onset, yr**	48.85 [±5.53]; 47.50 {39.00–57.00}
**FOG med-off condition** Absence Presence	11 (55.00%) 9 (45.00%)
**FOG med-on condition** Absence Presence	18 (90.00%) 2 (10.00%)
**UPDRS part I**	2.00 [±1.78]; 2.00 {0.00–8.00}
**UPDRS part II med-off condition**	19.50 [±5.75]; 20.00 {9.00–33.00}
**UPDRS part II med-on condition**	7.50 [±4.56]; 8.00 {1.00–17.00}
**UPDRS part-III med-off condition**	35.80 [±9.69]; 33.50 {25.00–62.00}
**UPDRS part-III med-on condition**	13.65 [±7.43]; 12.50 {3.00–31.00}
**UPDRS part IV**	6.79 [±2.15]; 7.00 {4.00–11.00}
**L-dopa responsiveness, % of improvement**	62.25 [±17.06]; 63.86 {34.00–93.94}
**Hoehn & Yahr med-off**	2.82 [±0.62]; 2.50 {2.00–4.00}
**Hoehn & Yahr med-on**	1.98 [±0.47]; 2.00 {1.00–2.50}
**UPDRS akinesia subscore med-off**	12.60 [±3.33]; 12.50 {7.00–18.00}
**UPDRS akinesia subscore med-on**	4.45 [±3.36]; 4.00 {0.00–12.00}
**UPDRS tremor subscore med-off**	4.60 [±4.31]; 3.00 {0.00–14.00}
**UPDRS tremor subscore med-on**	1.35 [±2.32]; 0.00 {0.00–9.00}
**UPDRS PIGD subscore med-off**	7.80 [±3.43]; 7.50 {3.00–16.00}
**UPDRS PIGD subscore med-on**	2.60 [±1.86]; 2.00 {0.00–7.00}
**LEDD (mg)**	913.26 [±398.289]; 1023.50 {200.00–1405.00}

Abbreviations: LEDD = L-dopa equivalent daily dose; MRI = magnetic resonance imaging; PD = Parkinson disease; PIGD = dominant postural instability and gait disorder; SD = standard deviation; WMH = white matter hyperintensities of vascular origin; UPDRS = Unified Parkinson’s Disease Rating Scale.

**Table A2:** Demographic and clinical characteristics at post-operative evaluation.

Variable	No. (%); Mean [±SD]; Median {Range}
**Age, yr**	63.40 [±5.60]; 64.00 {52.00–74.00}
**Disease duration, yr**	14.40 [±4.47]; 13.00 {8.00–28.00}
**Follow-up duration, yr**	4.55 [±1.32]; 5.00 {3.00–7.00}
**FOG stim-on/med-off condition** Absence Presence	11 (55%) 9 (45%)
**FOG stim-off/med-off condition** Absence Presence	7 (35%) 13 (65%)
**FOG stim-on/med-on condition** Absence Presence	15 (75%) 5 (25%)
**PD motor subtype** PIGD Indeterminate TD	17 (85%) 2 (10%) 1 (5%)
**UPDRS part I**	2.70 [±1.81]; 2.50 {0.00–7.00}
**UPDRS part II med-off condition**	18.60 [±5.73]; 19.50 {6.00–29.00}
**UPDRS part II med-on condition**	13.45 [±5.99]; 14.00 {4.00–23.00}
**UPDRS part-III stim-on/med-off**	29.10 [±13.71]; 23.00 {13.00–58.00}
**UPDRS part-III stim-off/med-off**	45.80 [±13.95]; 45.50 {25.00–73.00}
**UPDRS part-III stim-on/med-on**	15.80 [±9.73]; 12.50 {5.00–38.00}
**UPDRS akinesia subscore stim-on/med-off**	11.20 [±6.02]; 10.50 {2.00–23.00}
**UPDRS akinesia subscore stim-off/med-off**	17.25 [±6.50]; 17.50 {4.00–28.00}
**UPDRS akinesia subscore stim-on/med-on**	6.75 [±5.13]; 5.50 {0.00–17.00}
**UPDRS PIGD subscore stim-on/med-off**	7.55 [±3.25]; 7.50 {1.00–13.00}
**UPDRS PIGD subscore stim-off/med-off**	9.00 [±3.74]; 9.00 {1.00–15.00}
**UPDRS PIGD subscore stim-on/med-on**	4.80 [±3.71]; 4.50 {0.00–12.00}
**UPDRS tremor subscore stim-on/med-off**	3.05 [±2.61]; 3.50 {0.00–7.00}
**UPDRS tremor subscore stim-off/med-off**	4.70 [±3.15]; 4.00 {0.00–10.00}
**UPDRS tremor subscore stim-on/med-on**	0.60 [±1.05]; 0.00 {0.00–4.00}
**Hoehn & Yahr stim-on/med-off**	2.78 [±0.79]; 2.50 {2.00–5.00}
**Hoehn & Yahr stim-off/med-off**	3.55 [±1.09]; 3.50 {2.00–5.00}
**Hoehn & Yahr stim-on/med-on**	2.45 [±0.54]; 2.50 {2.00–4.00}
**Levodopa dose administered during the acute test, mg**	145.00 [±35.91]; 150.000 {100.00–250.00.}
**LEDD, mg**	828.65 [±372.861]; 799.500 {118.00–1500.00}
**Levodopa therapy duration, y**	11.80 [±2.88]; 12.00 {7.00–17.00}

Abbreviations: LEDD = L-dopa equivalent daily dose; UPDRS = Unified Parkinson’s Disease Rating Scale.

**Table A3:** Changes of neuropsychological variables.

Variables	Mean [±SD]; Median {Range}	
	Pre-Operative Assessment	Post-Operative Assessment
**Phonemic fluency**	33.51 [±7.77]; 33.37 {18.29–46.90}	27.11 [±11.29]; 27.14 {9.75–57.18}
**Spatial perception localization of numbers**	8.63 [±1.19]; 9.00 {7.00–10.00}	8.18 [±2.16]; 9.00 {3.00–10.00}
**1947 colored Raven’s progressive matrices**	28.83 [±4.61]; 30.47 {21.00–35.61}	25.30 [±6.04]; 24.48 {16.84–40.52}
**Stroop test “time”**	20.15 [±10.98]; 19.25 {8.00–47.00}	27.71 [±21.63]; 22.75 {6.50–91.50}
**Stroop test “errors”**	0.54 [±0.67]; 0.50 {0.00–2.25}	2.59 [±4.98]; 0.00 {0.00–15.75}
**Trail making test part B**	98.82 [±59.13]; 79.00 {33.00–274.00}	181.29 [±131.83]; 120.50 {33.00–531.00}

**Table A4:** Changes of FOG over time.

Variable	No. (%); Mean [±SD]; Median {Range}				
	Pre-Operative Assessment		Post-Operative Assessment		
	Off-Medication	On-Medication	On-Stimulation/Off-Medication	Off-Stimulation/Off-Medication	On-Stimulation/On-Medication
**Fog Score Variables**					
**Starting**	0.75 [±1.25]; ^§ 0.00 {0.00–3.00}	0.10 [±0.45]; ç° 0.00 {0.00–2.00}	0.50 [±1.05]; 0.00 {0.00–3.00}	0.95 [±1.36]; ^§ 0.00 {0.00–3.00}	0.15 [±0.49]; ç° 0.00 {0.00–2.00}
**Turning clockwise**	1.05 [±1.36]; ^§ 0.00 {0.00–3.00}	0.10 [±0.45]; ç*° 0.00 {0.00–2.00}	0.90 [±1.21]; ^°§ 0.00 {0.00–3.00}	1.07 [±1.34]; ^*§ 2.00 {0.00–3.00}	0.25 [±0.72]; ^*° 0.00 {0.00–3.00}
**Turning counter-clockwise**	1.05 [±1.36]; ^§ 0.00 {0.00–3.00}	0.10 [±0.45]; ç*° 0.00 {0.00–2.00}	1.00 [±1.21]; ^°§ 0.00 {0.00–3.00}	1.65 [±1.31]; ^*§ 2.00 {0.00–3.00}	0.25 [±0.72]; ç*° 0.00 {0.00–3.00}
**Doorway passage**	0.70 [±1.26]; ^ 0.00 {0.00–3.00}	0.10 [±0.45]; ç° 0.00 {0.00–2.00}	0.60 [±1.10]; ° 0.00 {0.00–3.00}	1.40 [±1.35]; ^*§ 2.00 {0.00–3.00}	0.25 [±0.79]; ° 0.00 {0.00–3.00}
**Total FOG score**	3.55 [±4.90]; ^°§ 0.00 {0.00–12.00}	0.40 [±1.23]; ç*° 0.00 {0.00–4.00}	3.05 [±4.06]; ^°§ 0.00 {0.00–12.00}	5.70 [±4.94]; ç^*§ 6.00 {0.00–12.00}	0.9 [±2.17]; ç*° 0.00 {0.00–9.00}

Abbreviations: LEDD = L-dopa equivalent daily dose; MRI = magnetic resonance imaging; PD = Parkinson disease; PIGD = dominant postural instability and gait disorder; SD = standard deviation; WMH = white matter hyperintensities of vascular origin; UPDRS = Unified Parkinson’s Disease Rating Scale. FOG variables: Friedman Test followed by Wilcoxon signed rank test post-hoc. * *p*-value < 0.005 with respect to the on-stimulation/off-medication condition. **°** *p*-value < 0.05 with respect to the off-stimulation/off-medication condition. § *p*-value < 0.05 with respect to the on-stimulation/on-medication condition. ç *p*-value < 0.005 with respect to the off-medication condition. ^ *p*-value < 0.05 with respect to the on-medication condition.

**Table A5:** Clinical characteristics between the “worsened” and “stable/improved” subgroups.

Variable	No. (%); Mean [±SD]; Median {Range}	
	“Worsened” (n = 5)	“Stable/Improved” (n = 15)
**Pre-operative variable**		
**UPDRS part-III on-medication**	27.80 [±7.29]; 23.00 {15.00–31.00}	10.60 [±4.47]; 11.00 {3.00–18.00}
**UPDRS akinesia subscore on-medication**	8.00 [±4.36]; 9.00 {1.00–12.00}	3.27 [±1.98]; 4.00 {0.00–07.00}
**Post-operative variable**		
**UPDRS akinesia subscore on stimulation/on-medication**	11.80 [±6.10]; 14.00 {2.00–17.00}	5.07 [±3.59]; 5.00 {0.00–12.00}
**UPDRS PIGD subscore on-stimulation/off-medication**	10.40 [±2.19]; 11.00 {7.00–13.00}	6.60 [±3.02]; 7.00 {1.00–12.00}
**UPDRS PIGD subscore off-stimulation/off-medication**	12.20 [±2.28]; 13.00 {9.00–15.00}	7.93 [±3.56]; 8.00 {1.00–13.00}
**UPDRS PIGD subscore on-stimulation/on-medication**	8.00 [±2.83]; 10.00 {4.00–10.00}	3.73 [±3.39]; 3.00 {0.00–12.00}
**Total FOG SCORE on-stimulation/off-medication**	7.00 [±4.30]; 9.00 {2.00–12.00}	1.73 [±3.10]; 0.00 {0.00–08.00}
