# Supplementary material for: Freezing of Gait in Parkinson’s Disease Patients Treated with Bilateral Subthalamic Nucleus Deep Brain Stimulation: A Long-Term Overview
Source: Biomedicines. 2022 Sep 7;10(9):2214. doi: 10.3390/biomedicines10092214 (PMC9496255; doi:10.3390/biomedicines10092214)
Supplement: Supplementary file 1 [file biomedicines-10-02214-s001.zip › biomedicines-1867925-supplementary.pdf]

Table S1. Stimulation parameters at post-operative long-term evaluation

Stimulation parameters and settings	Total *n*= 20<br>N. (%), mean, [±SD]; median {range}
**Frequency setting**<br>High frequency<br>Low frequency	 17 (85.00%)<br>3 (15.00%)
**Left STN**	
**Single monopolar stimulation**	15 (75.00%)
**Bipolar stimulation**	2 (10.00%)
**Double monopolar stimulation**	3 (15.00%)
**Voltage (V)**	2.640 [± 0.792];<br>2.800 {0.652-3.900}
**Frequency (Hz)**	132.500 [± 28.81];<br>130.000 {80.000-180.000}
**Pulse width (usec)**	64.500 [± 10.990];<br>60.000 {60.000 - 90.000}
**Impedance (Ω)**	937.150 [± 184.256];<br>945.500 {662.00 -1407.00}
**Power of stimulation**	66.535 [± 31.974];<br>69.302 {4.240-135.871}
**Right STN**	
**Single monopolar stimulation**	18 (90.00%)
**Bipolar stimulation**	0 (.00%)
**Double monopolar stimulation**	2 (10.00%)
**Voltage (V)**	2.466 [± 0.808];<br>2.650 {0.746-4.100}
**Frequency (Hz)**	123.400 [± 37.135];<br>130.000 {60.000-180.000}
**Pulse width (usec)**	69.500 [± 18.771];<br>60.000 {60.000-130.000}
**Impedance (Ω)**	964.850 [± 218.867];<br>951.500 {566.00 -1474.00}
**Power of stimulation**	56.599 [± 33.375];<br>50.022 {5.281 -121.571}
